# An Eco-Friendly Wood Adhesive Consisting of Soybean Protein and Cardanol-Based Epoxy for Wood Based Composites

**DOI:** 10.3390/polym14142831

**Published:** 2022-07-12

**Authors:** Zhiqiang Zhu, Erbing Zhang, Yijing Tu, Manyu Ye, Nairong Chen

**Affiliations:** College of Materials Engineering, Fujian Agriculture and Forestry University, Fuzhou 350002, China; mkkzq_zh@163.com (Z.Z.); 18726081410@163.com (E.Z.); qddxtyj@163.com (Y.T.); 15033575098@163.com (M.Y.)

**Keywords:** wood adhesive, soybean, epoxidized, water resistance, viscosity, plywood

## Abstract

Formaldehyde-derived wood adhesives have dominated in woody composites production up to now, while facing a significant challenge in non-renewable raw materials and the formaldehyde emission. To solve these problems, an eco-friendly soybean protein-based wood adhesive was explored via the addition of renewable cardanol based epoxy (CBE) as cross-linking agent. The curing mechanism and viscosity of the adhesives were investigated and the bonding performance was evaluated with three-ply plywood. Fourier transformed infrared spectroscopy (FTIR) analysis confirmed the formation of new ether linkages and the consumption of epoxy groups in the cured adhesives, thereby improving the thermal stabilities and cohesion. Plywood bonded with the CBE-modified soybean protein-based adhesive reached the maximum wet shear strength of 1.11 MPa (4 wt.% CBE addition), a 48% increase compared to the control, whereas the viscosity of adhesive decreased by 68.2%. The wet shear strength of the plywood met the requirements of the Chinese National Standard GB/T 9846-2015 for interior plywood application. The formaldehyde-free adhesive with excellent water resistance adhesiveness performance shows great potential in woody composites as an alternative to formaldehyde derived wood adhesives.

## 1. Introduction

The world is facing a petroleum resource crisis due to the growing energy demand and reduction of petroleum reserves, while fossil products have also brought about increasingly serious environmental pollution. Thus, the products derived from renewable and environmentally friendly biomass resources have received widespread attention. Natural wood is characterized by its sustainable and eco-friendly and has been widely used by human beings as a structural material for thousands of years in the world. Recently, natural wood has been widely replaced by wood-based composites (e.g., plywood) in interior and outdoor applications due to its weaknesses such as high water absorption, poor dimensional stability [1,2,3]. Adhesives/resins are a key factor in the manufacture of wood-based composites. Formaldehyde-based adhesives such as urea formaldehyde resin, urea-melamine formaldehyde resin, and phenol formaldehyde resin account for the 90% of current wood-adhesive market [4,5]. Formaldehyde has been classified by the World Health Organization as probably carcinogenic to humans [6]. The use of these adhesives for wood-based composites are not sustainable and can lead to environmental contamination and health issues for users [7,8]. Therefore, it remains a crucial challenge to seek a formaldehyde-free adhesive derived from biomass resources for wood-based composites.

Potential biomass including starch, protein, lignin, tannin, etc., are investigated for wood adhesives [4,9,10]. Among them, vegetable proteins are emerging as raw material for the preparation of biomass adhesives due to their numerous functional groups such as amino groups, carboxylic acid groups, and hydroxyl groups [11,12]. Soybean products such as defatted soybean flour, soybean protein isolate, which are characterized by their high protein content, low cost, and easily availability, are increasingly used as a raw material for the preparation of eco-friendly wood adhesives, but their commercial applications are restricted by poor water resistance [13,14]. Many strategies, such as the Maillard reactions between carbohydrate and protein, formation-hyperbranched structure, reinforcement with nano-particles, and cross-linking treatment with resins, have been made to modify the soybean products to obtain adhesives with the desired water resistance [15,16]. In particularly, petro-based resins as the cross-linking agents have received considerable attention due to the fact that they are widely successful in many wood-adhesive application [17,18]. Then, the combination containing renewable raw material and a cross-linking agent provides fully bio-based networks suggesting a new way to develop eco-friendly wood adhesives.

**Figure 1:**
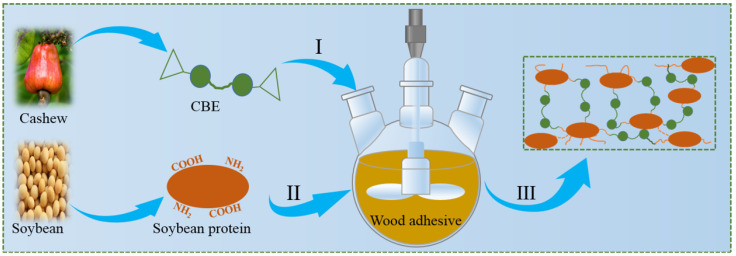
Schematic illustration of preparation soybean protein-based adhesive and its curing. (I: Ultrasonic dispersion: II: Add soybean protein and adjust pH; III: Hot-press curing).

Cardanol is a non-edible co-product from cashew-nut shell liquid-oil extracts, and is commercially available as epoxidized monomer or cardanol-based epoxy [19], which is well-known as a renewable material to produce thermoset resin with excellent properties [20]. Inspired by this, epoxidized cardanol provides the possibility of an ideal cross-linking agent for soybean-based wood adhesives. Thus, an eco-friendly wood adhesive with expected high water resistance performance was developed via a two-step process, as schematically shown in Figure 1. In the first step, epoxidized cardanol was dispersed into water to form an emulsion via an ultrasonic treatment. In the second step, soybean protein was added into the emulsion and further adjusted to pH 11 using sodium hydroxide to obtain the eco-friendly wood adhesives. Sodium hydroxide acts as a modifier to unfold the quaternary and/or tertiary structure of soybean protein, and expose its functional groups in the globular structure to outside [21], because the protein often has polar groups buried inside the globular and nonpolar groups on the outside. Polar groups such as carboxyl, hydroxyl, and amino are the key chemical reaction functional groups for increased cross-linking structure [11]. The aim of this work was to evaluate and characterize an eco-friendly wood adhesive derived from the sustainable materials, soybean protein and epoxidized cardanol, for wood composite application. Typically, its adhesion performance after water-soaking treatment was evaluated. The adhesive was characterized by Fourier transform infrared spectroscopy (FTIR), scanning electron microscopy (SEM), differential thermal gravity (DTG) and thermogravimetric (TG) analyses, rotational rheometer, and then applied to the production of plywood for shear strength test.

## 2. Materials and Methods

### 2.1. Materials

Soybean protein isolate (protein content ≥ 95%) and defatted soybean flour with 90% protein dispersibility index were purchased from Shandong Wonderful Industrial Group Co., Ltd. (Kenli, China). Cardanol-based epoxy (CBE, epoxy equivalent is 350–500 g/eq) was purchased from Cardolite Chemical Zhuhai Co., Ltd. (Guangzhou, China). Sodium hydroxide (analytical grade) and potassium bromide (spectrally pure) was purchased from Sinopharm Chemical Reagent Beijing Co., Ltd. (Beijing, China). Eucalyptus veneers (300 mm × 300 mm, 1.7 mm thickness, moisture content 10–12%) were supplied by Wenshan Tongxuan Wood Industry Co., Ltd. (Yunnan, China).

### 2.2. Soybean Protein-Based Adhesive Preparation

CBE with different additive amounts (Table 1) was added into deionized water and stirred for 30 min at 35 °C. The obtained CBE dispersion was then stirred and sonicated in an ultrasonic cleaner for 10 min at 25 °C to form a homogeneous emulsion. Next, soybean protein isolate (15 g) was slowly added into the emulsion and continously stirred for another 15 min. Finally, the pH of the slurry was adjusted to 11 by 30% (mass fraction) sodium hydroxide solution to obtain the CBE-modified soybean protein-based adhesives.

### 2.3. Characterization

CBE-modified soybean protein-based adhesive was oven dried at 120 °C for 2 h to obtain the cured adhesive samples for further characterization. Fourier transform infrared spectroscopy (FTIR) was performed with a Nicolet 380 FTIR spectrometer (Thermo Fisher Scientific, Waltham, MA, USA), over a region of 400–4000 cm^−1^. The cured adhesive samples (0.001 g) were ground into a powder, and mixed with potassium bromide (0.1 g), and then pressed under a pressure of 18 MPa for 30 s. The software Nicolet OMNIC V 8.2 was employed for spectra analysis. The thermal stability analysis of the cured adhesive samples was carried out on a NETZSCH STA449F3 TGA instrument (NETZSCH Co., Selb, Germany) with NETZSCH Proteus software V 5.2.1. Approximately 6.6 mg of powdered adhesive samples were weighed and scanned from 30 °C to 800 °C at a heating rate of 10 °C/min in a nitrogen environment. A Nova NanoSEM 230 (FEI CZECH REPUBLIC S.R.O, Brno, Czech Republic) was used to observe the surface of the cured adhesives, which were coated with 10 nm Au/Pd film to get a good conductivity, and the different surface morphology was analyzed.

The viscosity of the CBE-modified soybean protein-based adhesives was determined on a Hacker rheometer (Thermo Fisher Scientific, Waltham, MA, USA), and using a PP35Ti parallel plate with a gap of 0.105 mm. The shear stress (0~400 γ/s^−1^) and apparent viscosity at different shear rates were measured at 25 °C.

**Figure 2:**
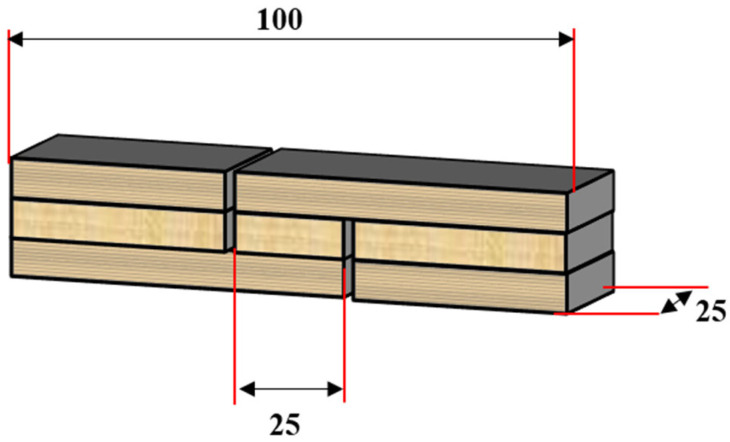
The specimen of plywood for shear strength test (Units: mm).

The adhesiveness performance was assessed by wet shear strength of plywood bonded with the CBE-modified soybean protein-based adhesives. The three-layer plywood was prepared by brushing about 340 g/m^2^ of the adhesive on the double-side of one veneer. The coated veneer was placed as a middle layer, then covered by the other two un-coated veneers with grain vertical to the middle veneer, followed by hot-pressing with a temperature of 120 °C, pressure 1 MPa, and time 180 s. After hot-pressing, three-layer plywood was conditioned in a ventilated environment for 48 h. Shear strength of the plywood measured in accordance with the Chinese National Standards GB/T 17657-2013. Each plywood was cut into 10 pieces of specimens (Figure 2), which were soaked into a 63 °C water bath for 3 h, and then cooled to room temperature for 10 min. After that, the specimens were tested in a tensile-testing machine (MTS, Shenzhen, China) with a crosshead speed of 5.0 mm/min. Two replicates were used for each adhesive.

## 3. Results and Discussion

**Figure 3:**
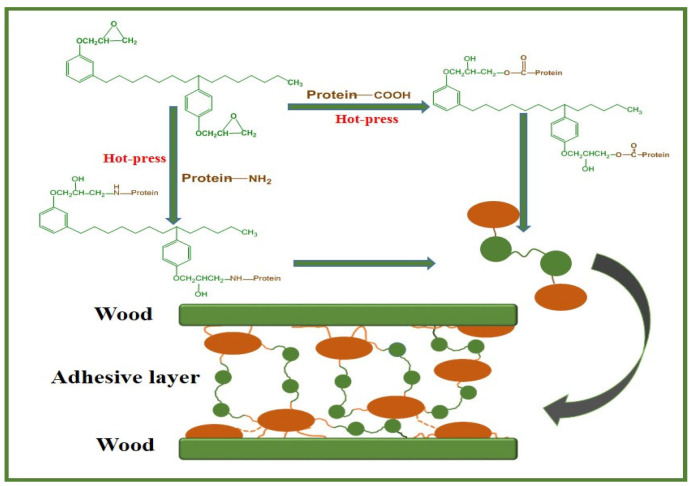
Possible reaction in the curing process of soybean protein-based adhesives.

The main reactive functional groups in soybean protein are carboxyl groups (–COOH), amino groups (–NH_2_), and hydroxyl groups (–OH), which are readily cross-linking to epoxy groups under a high temperature [22]. A possible chemical reaction process is shown in Figure 3. In this way, various complex tightly-linked structures may be formed during the curing process of the adhesive, which reduce its defects, such as cracks and holes, thereby improving the water resistance and shear strength of plywood [13].

### 3.1. FTIR Analysis

**Figure 4:**
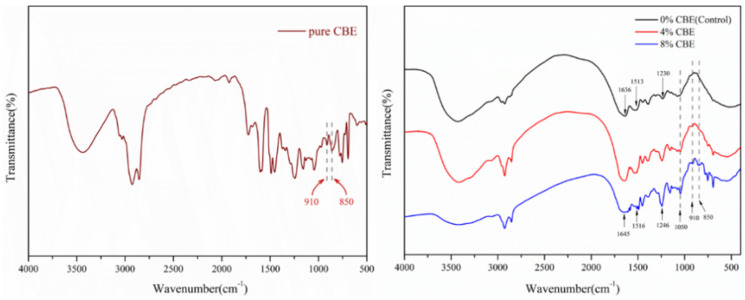
FTIR spectra of CBE and soybean protein-based adhesives with different CBE content.

To characterize the variation of functional groups in the adhesives with CBE, FTIR analysis was performed. As showed in the Figure 4, the typically characteristic peaks of epoxy group could be found at 850 cm^−1^ and 910 cm^−1^ in the FTIR spectrum of pure CBE [23,24]. In the control sample (0% CBE), the peaks of the soybean protein at 1636 cm^−1^, 1513 cm^−1^, and 1230 cm^−1^ were characteristic of amide I (C=O stretching), amide II (N-H bending), and amide III (C-N and N-H stretching), respectively. After adding CBE to adhesive, the amide bonds had significant blue shift (shifted to 1645 cm^−1^, 1516 cm^−1^, and 1246 cm^−1^ in the 8% CBE). This indicated that the bond vibration required more energy in the adhesive, which means the cross-linking structure was formed [5]. Compared with the control sample (0% CBE) spectrum, a new peak at 1050 cm^−1^ in the sample 4% CBE was observed which was attributable to ester bonds and the characteristic peak of the epoxy group almost disappeared at 850 cm^−1^ and 910 cm^−1^. These suggested that the epoxy group in CBE reacted with polarity groups in soybean protein to form the ether linkage and further form a cross-linked structure [2]. In addition, the epoxy group peaks (850 cm^−1^ and 910 cm^−1^) were observed in 8% CBE. It indicated that 8% of CBE was too much for cross-linking soybean protein. The reactions between soybean protein and CBE will form a three-dimensional cross-linking structure, resulting in the improvement of mechanical properties and wet-cohesion of the cured CBE-modified soybean protein-based adhesives [25,26]. However, the residual CBE in the cured adhesive might have acted as a plasticizer to decline its mechanical properties.

### 3.2. Thermal Stability Analysis

**Figure 5:**
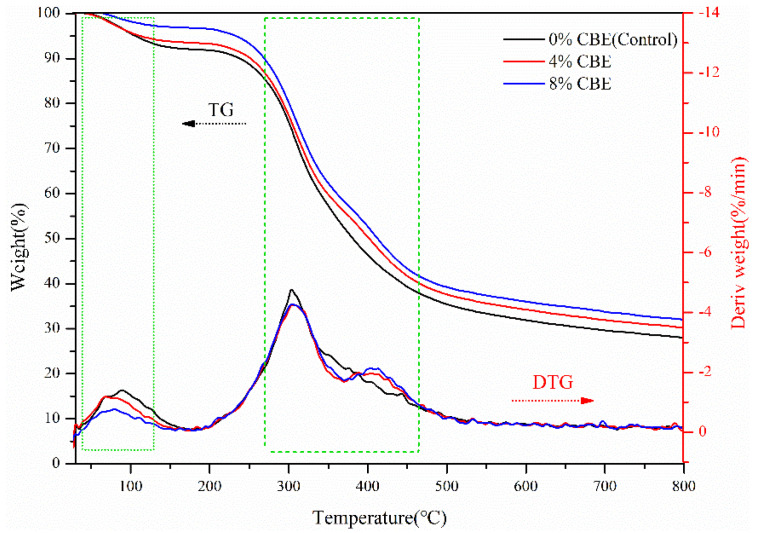
TG/DTG curves of soybean protein-based adhesives with different CBE content.

The thermal stabilities of the cured CBE-modified soybean protein-based adhesives were tested and analyzed by using Differential thermal gravity (DTG) and Thermogravimetric (TG) analyses. The TG and DTG curves of the adhesive samples showed the two obvious thermal weight-loss processes (Figure 5). The first stage occurred at 30 °C to 175 °C, which was ascribed to the escape of small molecules and water in the soybean protein components [8]. In the second stage (175 °C to 550 °C), soybean protein components were thermally decomposed fast with obvious weight loss (e.g., the control) [27]. The main reason could be the low cross-linking density between the amino-acid residuals, which lead to the poor thermal stability [28], whereas, the cross-linked adhesive sample was more stable, thus requiring the higher degradation temperature. When the temperature was about 550 °C and beyond, the carbonization residue was degraded into CO_2_ and H_2_O, and the mass of the adhesive samples changed little.

### 3.3. Morphology Analysis

**Figure 6:**
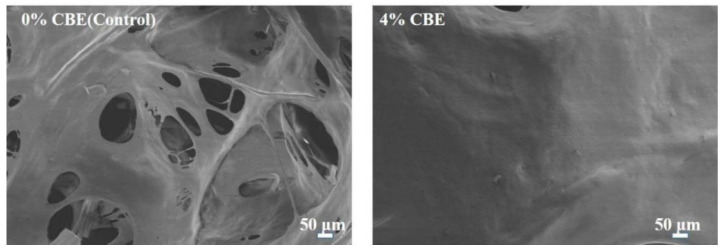
SEM images of fracture surfaces of the cured soybean protein-based adhesives.

SEM was employed to observe the surface morphology features of cured adhesives. Different pore structures could be found in the SEM images (Figure 6), which was mainly caused by the increased gasification pressure and volume of water in the curing process of sample adhesives. The cured CBE-modified soybean protein-based adhesive samples had better compact surface and less pores than the control, suggesting that CBE can improve cohesion of the pure soybean-protein adhesive [29]. It could be attributed to the three-dimensional cross-linked structure of the CBE-modified adhesive having better mechanical performance to resist the damage of water gasification in the curing process.

### 3.4. Rheological Analysis

**Figure 7:**
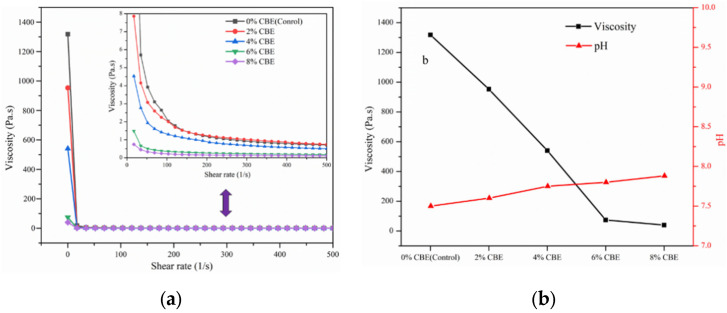
Rheological curves (**a**), initial viscosity and pH (**b**) of soybean protein-based adhesive with different CBE content.

The rheological properties of soybean protein-based adhesives with different CBE content were also investigated. Figure 7a showed the relationship between the viscosity and the shear rate of soybean protein-based adhesives. The viscosity of soybean protein-based adhesives decreased as the shear rate increased. It was indicated that the adhesives with or without CBE are the non-Newtonian fluid with shear thinning behavior [30]. At a consistent shear rate, the viscosity had followed a similar decreased tendency as the content of CBE increased, i.e., a sharp decrease in the initial viscosity was observed in the adhesives with CBE addition, while the pH of the adhesives fluctuated only within ± 0.25 (Figure 7b), which probably was caused by the CBE with a small molecular weight. It is reported that the molecules with small molecular weight can play a role as the lubricant/plasticizer to decrease viscosity in the system. Usually, low viscosity can facilitate the spreading, flow, and penetration of the adhesives into the wood and further form the cured adhesive−wood mechanical interlocking to improve shear strength significantly [31,32].

### 3.5. Adhesiveness Analysis

**Figure 8:**
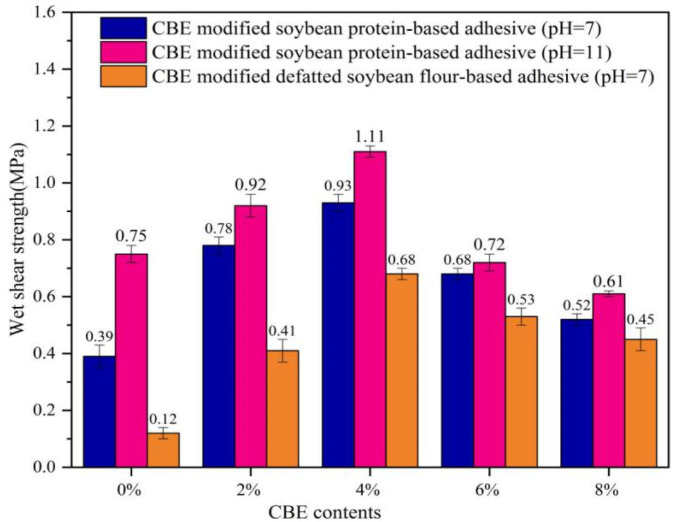
Effect of CBE content on wet shear strength of soybean-based adhesive.

It is well known that a higher wet shear strength of plywood can be achieved by the cross-linking structure, cohesion, and water resistance of the cured wood adhesive [31,33]. Hence, three-ply plywood was prepared to investigate the adhesiveness of soybean protein-based adhesive according to the Chinese National Standards GB/T 17657-2013. Figure 8 showed the wet shear strength of plywood bonded with CBE modified soybean-based adhesives. The wet shear strength increased as CBE content (the adhesive with pH 11) was increased, and then decreased at CBE content of approximately 4% and beyond, implying that the CBE content should be less than 4%, because the un-reacted CBE may act as a plasticizer to decline the glass transition temperature of cured adhesive, resulting to the poor adhesiveness [34]. The maximum wet shear strength was 1.11 MPa when the CBE content was 4%, which represented that it increased by 48% relative to the control sample. The result was higher than the requirement of the Chinese National Standard GB/T 9846-2015 for interior plywood (≥0.7 MPa). In order to further verify the cross-linking ability of CBE, the adhesive with different pH (pH = 7) or raw material (defatted soybean flour) was prepared. The results showed the similar trend to plywood bonded with CBE modified soybean protein-based adhesive (Figure 8). However, the adhesiveness of all the adhesives at pH 11 was higher than that at pH 7, which was caused by the high level of exposure of the functional groups of soybean protein at pH 11, leading to the higher cross-linking density of cured adhesives [35,36]. Compared to the adhesiveness of defatted soybean flour-based adhesive, all the CBE-modified soybean-based adhesive displayed better performance. It could be ascribed to the poor purity of defatted soybean flour, which contained about 40% carbohydrates, leading to the poor water resistance. Nevertheless, when 4% of CBE was added into the soybean-based adhesive, the wet shear strength of the plywood still met the requirements of the Chinese National Standard GB/T 9846-2015 for interior plywood. Therefore, CBE is an efficient bio-based cross-linking agent for improving the bonding performance of soybean protein-based adhesives.

## 4. Conclusions

An all biomass-based, formaldehyde-free, and eco-friendly wood adhesive composed of soybean protein isolated, cardanol-based epoxy, sodium hydroxide, and water was developed in this study. Cardanol-based epoxy acted as a cross-linking agent for reacting with hydrophilic groups in soybean protein. Sodium hydroxide unfolded the globular soybean protein and exposed its hydrophilic groups inside to outside, and reacted with cardanol-based epoxy, which improved the cross-linking density, flatness, thermal stability of the cured adhesive, providing better mechanical properties for final wood-based composites. The viscosity of the adhesives decreased as the shear rate and/or cardanol based epoxy content increased. When the adhesive was modified by 4% of cardanol-based epoxy, the plywood displayed the maximum wet shear strength, which met the requirements of the Chinese National Standard GB/T 9846-2015 for interior plywood. Cardanol-based epoxy is an effective cross-linking agent for vegetable protein biomass to develop the ‘green’ wood adhesives.

## Figures and Tables

**Table 1:** Formulations of the adhesives with different CBE content.

CBE Content Ratio (%)	SPI (g)	Water (g)	CBE (g)
0 (Control)	15	85	0
2	15	83	2
4	15	81	4
6	15	79	6
8	15	77	8

## Data Availability

Not applicable.
